# Outcome of open and endovascular repair in acute type B aortic dissection: a retrospective and observational study

**DOI:** 10.1186/1749-8090-5-23

**Published:** 2010-04-09

**Authors:** Pasquale Mastroroberto, Francesco Onorati, Saverio Zofrea, Attilio Renzulli, Ciro Indolfi

**Affiliations:** 1 Department of Experimental and Clinical Medicine, Cardiovascular Surgery Unit University Magna Græcia, viale Europa, 88100 Catanzaro, Italy; 2 Department of Experimental and Clinical Medicine, Cardiology Unit University Magna Græcia, viale Europa, 88100 Catanzaro, Italy

## Abstract

**Background:**

The aim of the study was to analyze surgical and endovascular results in the treatment of acute type B aortic dissection (B AAD).

**Methods:**

Retrospective and observational analysis with patient inclusion between January 2001-December 2008 and follow-up ranged from 2 to 96 months (median = 47.2) was performed. Out of 51 consecutive patients with B AAD, 11 (21.6%) had to undergo open surgery (OS) and 13 (25.5%) endovascular treatment (TEVAR).

**Results:**

There was a significantly difference in early mortality in the TEVAR group (0/13,0%) vs OS group (4/11,36.4%, P < 0.05) and in the incidence of paraplegia/paraparesis (OS 2,28.6% vs TEVAR 1,7.7%, P < 0.05), renal failure (OS 3, 42.8% vs TEVAR 1, 7.7%, P < 0.05), respiratory failure (OS 2,28.6% vs TEVAR 1,7.7%, P < 0.05) and cerebrovascular accident (OS 1,14.3% vs TEVAR 0,0%, P < 0.05). The late mortality at a follow-up was 30.8% (4/13) in the TEVAR group and 42.8% (3/7) in the OS group, respectively (P = not significant). The cumulative survival rate after 1, 3 and 8 years was 93%, 84%, and 69% in the TEVAR group and 86%, 71% and 57% in the OS group, respectively. Endoleaks were diagnosed in 2/13 endovascular patients (15.4%).

**Conclusions:**

TEVAR group had a significantly reduction in early mortality and postoperative complications. No significant differences were found in terms of cumulative survival at follow-up. On this basis TEVAR could be considered an option in the treatment of these complex cases with all proper reservation especially related to the small sample sizes examined.

## Background

The treatment of Stanford type B acute aortic dissection (B AAD) still remains a formidable challenge in complicated cases and the options are medical therapy, conventional surgery or endovascular repair. The method of choice is conservative with aggressive medical therapy [1,2] using β- blockers, calcium-channel blockers and nitroglycerin to control heart rate and to maintain a systolic blood pressure less than 110 mmHg so lowering aortic wall tension. A surgical approach is reserved in all cases with complicated course such as persisted pain, rupture or impending rupture, visceral and/or leg ischemia with a mortality rate up to 50% [3] and high paraplegia rate [4], despite improved surgical techniques and perioperative care [5]. The recent review presented by the International Registry of Acute Aortic Dissection (IRAD) shows a surgical mortality of 27.8% and 62.5% in patients with malperfusion and rupture, respectively [6]. On this basis the application of thoracic endovascular aortic repair (TEVAR) has been introduced as alternative surgical option but its role remains to be debated with controversial opinions [7]. Many studies have examined a heterogeneous population of patients including acute and chronic type B aortic dissection with immediate versus delayed treatment and both complicated and uncomplicated cases. For this reason, the purpose of this study was to compare our surgical and endovascular results in the treatment of complicated B AAD and the patients enrolled were included in a multidisciplinary program called Magna Græcia AORtic Interventional Project^® ^* (MAORI^2002^).

* = *The Magna Græcia AORtic Interventional Project^® ^is a non-profit registered mark on the initiative of the first Author, PM. The activity is related to clinical, technological and scientific research on the diseases of the aorta. A collaborative multidisciplinary team consisting of cardiovascular surgeons, interventional cardiologists, radiologists, anaesthetists, radiologists, geneticists, nephrologists is involved in the program*.

## Methods

A consecutive series of 51 patients with B AAD were admitted to our large community University Hospital from January 2001 to December 2008. B AAD was defined as nontraumatic dissection involving the descending aorta with initial intimal tear distal from the origin of the left subclavian artery. Diagnosis was performed in all patients with both echocardiography and computed tomography (CT). Indications for a non-medical treatment was determined by rupture, high suspicion of impeding aortic rupture and visceral and/or peripheral ischemia based on clinical evaluation and CT imaging in the acute phase (within 14 days after the onset of symptoms). The signs of impending rupture were determined by persisting pain despite adequate and aggressive medical therapy, evidence of aortic expansion and presence of new ulcerlike CT projection. Organ and/or limb malperfusion were defined on the basis of clinical symptoms, physical examination and imaging detection. All patients received medical therapy as standard protocol with β-blockers, nitroglycerin and, in the last years, fenoldopam mesylate, a dopamine D_1 _- like receptor agonist used to control blood pressure and to obtain an optimal visceral perfusion. In all cases continuous arterial pressure monitoring, central venous cannulation for administration of all medications and urine output monitoring were performed. Pain resolved with controlled blood pressure and analgesia with absence of signs of malperfusion was observed in 27 (52.9%) and these cases were considered as treated only with medical therapy.

The surgical patients were divided in two groups, open surgery (OS) group with 11 patients (21.6%) and TEVAR group with 13 patients (25.5%), and all data were retrospectively analyzed. From 2002, considering surgical high mortality rate, our treatment was addressed also to the endovascular approach especially in high-risk cases and all patients excluded from this therapy were operated on conventional surgery. The specific technical and clinical criteria of exclusion from TEVAR will explained in a followed section ("TEVAR technique description"). The preoperative characteristics including the life-threatening complications of the two groups are listed in Table 1.

**Table 1:** Preoperative characteristics in the two groups of patients

*Variables*	*OS group* *No (%) or mean ± SD*	*TEVAR group* *No (%) or mean ± SD*	*P Value*
Patient total	11	13	

Age, years	70.2 ± 7.8	74.3 ± 8.4	< 0.05

Sex			
Male	8 (72.7)	7 (53.8)	NS
Female	3 (27.3)	6 (46.2)	NS

Hypertension	9 (81.8)	10 (76.9)	NS

Diabetes	2 (18.2)	1 (7.7)	NS

Serum creatinine (mean ± SD mg/dL)	1.2 ± 0.5	1.3 ± 0.4	NS

CAD	1 (9.1)	1(7.7)	NS

COPD	7 (63.6)	8 (61.6)	NS

Previous CVA	2 (18.2)	1 (7.7)	NS

Prior AAA repair	1 (9.1)	1 (7.7)	NS

Signs of aortic rupture	3 (27.3)	4 (30.8)	NS

AAA = abdominal aortic aneurysm; CAD = coronary artery disease; CVA = cerebrovascular accident; COPD = chronic obstructive pulmonary disease; OS = open surgery; SD = standard deviation; TEVAR = thoracic endovascular aortic repair; NS = not significant

Data from early mortality and postoperative complications as paraplegia or paraparesis, renal and respiratory failure, myocardial infarction, ventricular arrhythmias, congestive heart failure were also collected.

Follow-up data were obtained by retrospective reviews and clinic visits and CT scan performed at 3, 6 and 12 months after aortic repair and annually thereafter, and late survival rates do not include early mortality.

### Operative technique in the OS group

In all patients the graft replacement between the distal aortic arch and the descending thoracic aorta was performed through a left posterolateral thoracotomy as previously described [8] preparing proximal and distal aortic cuffs using biological glues (gelatine - resorcine - formaldehyde, the socalled "French glue", and recently a two - component adhesive composed of purified bovine serum albumin and glutaraldehyde - BioGlue^®^, CryoLife Inc, Kennesaw, GA, USA) and external strips of Teflon (Impra Inc, subsidiary of L.R. Bard, Tempe, AZ, USA) felt to reinforce the wall. 9 patients (77.8%) required the replacement of proximal half of descending aorta and 2 patients (22.2%) needed repair also in the distal half. Briefly our surgical approach involved cerebrospinal fluid drainage and perfusion of the distal aorta. All patients were positioned on the operating table in the lateral position with the abdomen and the pelvis turned so that the groin was at a 45° angle to the table to allow cannulation of the femoral vessels for partial femoro-femoral extracorporeal circulation. The proximal clamp was placed just below the origin of left subclavian artery in 9 patients and between the left carotid artery and left subclavian artery in 2 cases. The presence of a dissected aorta was considered contraindication to intercostal artery reimplantation. A cerebrospinal fluid catheter was inserted before the operation at the level of L3 or L4, and a pressure of 10 mm Hg or below was maintained. This pressure was monitored for 48 hours after the operation in the absence of lower extremity deficits. The drainage catheter was reinserted if a neurologic deficit developed after this period.

### TEVAR technique

The option of TEVAR as first therapeutic approach in cases with complicated type B AAD was considered because of a) enhanced experience of our multidisciplinary team, b) patients who were deemed a high-risk open-repair candidate (age ≥ 75 years-old, severe chronic obstructive pulmonary disease, serum creatinine ≥ 1.5 mg/dL, coronary artery disease with/without prior coronary artery surgery, congestive heart failure), c) favorable anatomic characteristics for TEVAR determined by the cardiovascular surgeon and interventional cardiologist. Patients presenting a landing zone < 1.5 cm with need to cover critical branch vessels, severe calcification at the fixation site of the graft, significantly tortuous and inadequate access vessels were excluded from TEVAR. The procedures were done with local or general anaesthesia using in all patients transesophageal echocardiography (TEE) to visualize the correct placement of the endoprosthesis, to achieve wire access in the true lumen before stent graft deployment and to confirm the exclusion of the false lumen. A cerebrospinal fluid catheter was also inserted before the procedure to detect neurologic events as spinal cord ischemia due to sustained hypotension during stent-graft placement or to coverage of major medullary arteries. The endograft delivery was performed via femoral artery open access and the other femoral artery or the right brachial artery were used to obtain necessary angiograms. In all patients we used the Talent™ endoluminal stent-graft system (Medtronic Vascular Inc., Sunrise, FL, USA) and balloon dilatation was not performed to prevent retrograde type A aortic dissection and/or aortic rupture [9]. In 3 patients (23%) the aortic coverage extended just above the origin of the celiac artery without its coverage using 2 stent-grafts, whereas in the other 10 patients (77%) one device was used to achieve adequate distal seal zone. Technical success of TEVAR was considered the placement of patent endograft, exclusion of the false lumen and absence of type I or III endoleaks.

### Statistical analysis

Data were analyzed with the SPSS software version 15.0 for Windows (SPSS Inc, Chicago, IL, USA). Continuous variables were presented as mean ± SD and categorical variables as frequency and percentage. Categorial variables were compared using the Fisher's exact test. Student's t-test was used to compare normally distributed continuous variables and Mann-Whitney U-test for variables without normal distribution. A two-tailed P value of less than 0.05 was considered to be statistically significant. Survival was analyzed with the Kaplan-Meier method [10] and was expressed as a percentage ± Standard Deviation (SD).

## Results

All patients medically treated were discharged from the hospital without any complications.

As reported in Table 1 the TEVAR group was older and had a higher but not significant frequency of signs of aortic rupture. The incidence of chronic obstructive pulmonary disease (COPD) was similar in the two groups (P = not significant) and preoperative comorbidities as hypertension and diabetes were slightly higher but not significants in TEVAR and OS group, respectively. The mean time from onset of symptoms to operation was 4.2 ± 2.1 days in all patients, independently from the type of procedure.

### TEVAR group (Table 2)

**Table 2:** Complications in survived patients of both the OS and TEVAR group

*Complication*	*OS group* *No(%)*	*TEVAR group* *No (%)*	*P value*
*No of patients*	*7/11*	*13/13*	

Paraplegia/paraparesis	2 (28.6)	1 (7.7)	<0.05

Renal failure	3 (42.8)	1 (7.7)	<0.05

Respiratory failure	2 (28.6)	1 (7.7)	<0.05

Cardiac	1 (14.3)	1 (7.7)	<0.05

Cerebrovascular accident	1 (14.3)	0 (0)	<0.05

Bleeding	1 (14.3)	0(0)	<0.05

Reinterventions	0 (0)	2(15.4)	<0.05

OS = open surgery; TEVAR = thoracic endovascular aortic repair

Technical success of TEVAR was achieved in all patients and the exclusion of false lumen and the absence of endoleaks were confirmed by TEE. General anaesthesia was reserved in only 2 cases (15.4%) because of haemodynamic instability. The left subclavian artery was crossed with the uncovered portion of the stentgraft in eight cases and the covered segment in the other five patients without subclavian- to - carotid bypass intervention.

The early mortality defined as either in-hospital or within 30 days was 0/13 (0%). The incidence of paraplegia/paraparesis, renal failure, respiratory failure with prolonged intubation and cerebrovascular accident were significantly lower than patients of the OS group. One patient emerged from anaesthesia neurologically intact but signs of significant lower extremity paresis were evident 20 days after the procedure probably due to delayed occlusion of a major medullary artery. No access-related complications in the TEVAR group were documented.

The postoperative length of stay was significantly shorter than the OS group at a median of 6 days versus 16 days. (P < 0.05).

**Figure 1:**
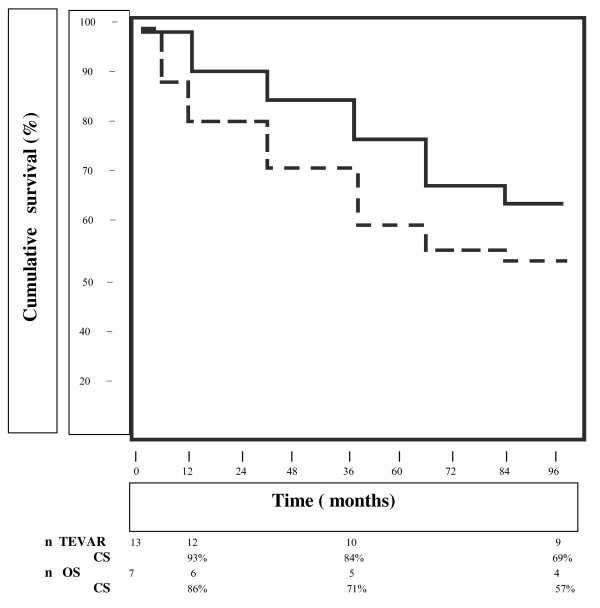
**Comparative survival analysis of OS (dashed line) and TEVAR (solid line) groups by Kaplan-Meier method**.

The late mortality at a follow-up, ranged from 2 to 96 months (median = 47.2), was 30.8% (4/13). The cumulative survival rate after 1, 3 and 8 years was 93%, 84%, and 69% (Fig. 1). Two endoleaks (15.4%) were revealed by CT scan at two and six months, respectively, one proximal endoleak probably due to poor seal of the graft was resolved by balloon dilatation and one distal endoleak was treated by an adjunctive stent-graft. On this basis a p value < 0.05 was found comparing results between OS and TEVAR groups in terms of late reinterventions.

### OS group (Table 2)

#### Early mortality was 36.4% (4 patients/11)

One intraoperative death (9.1%) occurred in a patient who had a dramatic anterolateral aortic rupture, 1 perioperative death (9.1%) was related to an anteroseptal myocardial infarction, 2 patients (18.2%) presented multiorgan failure and in 1 (9.1%) patient the postoperative course was complicated by renal failure and extensive bowel infarction.

Complications of the 7 surviving patients are summarized in Table 2. Paraplegia/paraparesis was seen in 2 cases (28.6%): one case of paraparesis, defined as weakness of both legs, was completely resolved six months after the operation while one case of paraplegia, defined as paralysis of both legs, was unresolved at follow-up. Renal failure needing haemodialysis occurred in 2 patients and one patient presented left hemiplegia because of cerebrovascular ischemic accident diagnosed by CT scan. Moreover one patient was reoperated on because of perianastomotic bleeding and required additional suture and external Teflon felt. The late mortality was 42.8% (3/7) and the cumulative survival rate after 1, 3 and 8 years was 86%, 71% and 57% (Fig. 1). No late surgical complications as pseudoaneurysm, infection of the grafts or fistulae from the graft to adjacent organ were diagnosed in this group.

## Discussion

Type B AAD is not considered as life-threatening as acute type A aortic dissection and medical management must be preferred with a low mortality rate [5,11]. These results are confirmed also in our series of medical-treated patients with no cases of hospital mortality. Patients with life-threatening complications as rupture, signs of impeding rupture as new ulcerlike projection, expanding false lumen or persistent symptoms and visceral and/or limb malperfusion are at high risk and require a more aggressive approach representing a very clinical challenge. In this setting TEVAR, open surgical aortic graft replacement, flap fenestration by catheterization or conventional surgery, extra-anatomic surgical bypass have been proposed as treatment in an emergency fashion. The primary objective of both OS and TEVAR approaches are obviously strictly related to reduce the risk of death and to minimize the complications by excluding proximal intimal tear, removing associated aneurysmal disease and maintaining a complete distal perfusion. The issuing advent of endovascular procedures has extended the number of patients potentially suitable for aortic repair by a minimally invasive option with a reduction in terms of mortality and morbidity [12-14] but some limitations have been questioned by the Expert Consensus Document recently published [7]. These limitations as the low probability to eliminate all flow in the false lumen, the remaining risk for late aneurysmal degeneration and aortic rupture are referable to chronic type B aortic dissection and must be considered quite reasonable. Based on the INSTEAD (INvestigation of STEnt grafts in patients with type B Aortic Dissection) results reported in the Expert Consensus Document [7] it is evident that TEVAR approach appears adequate in the early postoperative phase but presents no benefits when the risk of late aortic rupture and the life expectancy are examined [7]. The increasing relevance of thoracic endovascular repair has been demonstrated in a recent paper by Patel and coworkers [14] especially in a group of TEVAR patients older and sicker than patients scheduled for conventional surgery so the Author concludes that "The differences between the groups (endovascular and conventional surgery) therefore only serve to strengthen our conclusion that TEVAR should be the therapeutic option of choice in the elderly patient population" (see "Panel Discussion" at the end of the paper). Similarly our TEVAR group is significantly older than OS group so that endovascular approach could be considered the treatment of choice in a subset of patients considering age a risk factor in terms of morbidity and mortality. Type B AAD is quite another matter so the primary goal is to reduce early mortality and on this way TEVAR treatment is a valuable application if used with propriety of indications [7]. Our results confirm this tendency with a significantly difference between the TEVAR group vs the OS group especially in terms of early mortality (0% vs 36.4%, P < 0.05) and postoperative hospital stay (P < 0.05). In any case the analysis of causes of hospital mortality revealed that in the OS group one patient died from acute myocardial infarction and one from renal failure and bowel infarction so we concluded that probably these deaths are strictly related to a preoperative status of coronary artery disease and to a dramatic evolutive dissecting process respectively and were independently from the technique used (OS vs TEVAR). In the analysis of postoperative complications as paraplegia, renal and respiratory failure, cardiac arrhythmias and cerebrovascular accident a difference in favour of the TEVAR group was also found (Table 2). Moreover these complications appear to be strictly related to the procedure itself but not determined by possible preoperative risk factors as age. Several studies have documented significant morbidity correlated to TEVAR for type B AAD as acute or delayed retrograde type A dissection [15], paraplegia[16], stroke [16], access-related complications [17], endoleaks [16], visceral ischemia [16]. In our series of patients undergoing endovascular treatment no retrograde type A dissection, stroke, visceral ischemia and access-related complications were diagnosed. The incidence of paraplegia (1/13, 7.7%) was less in the TEVAR than in the OS group with a percentage of endoleaks of 15.4% (2/13). As previously reported [18] we believe that the high incidence of a catastrophic complication as retrograde type A dissection [9] may be prevented by the use of a stentgraft with an appropriate size not requiring balloon dilatation and paying attention that the guide wire is not misplaced in the false lumen.

The potential advantage of TEVAR therapy in all patients in the acute phase of type B aortic dissection with life-threatening complications probably fails in stable dissection. Recently Nienaber and coworkers [19] on behalf of the INSTEAD study group presented the results in one hundred forty patients in stable condition after at least two weeks the diagnosis of type B AAD, randomly assigned to elective TEVAR in addition to medical therapy or to optimal medical therapy alone. The 2 - year cumulative survival rate was 95.6 +/- 2.5% in patients with optimal medical therapy versus 88.9 +/- 3.7% in the TEVAR group and no difference was found regarding the aorta-related death rate. Moreover the comparison between the aortic rupture rate and the progression was similar in the two groups with a significantly difference only in the aortic remodelling represented by the true- lumen recovery and the false-lumen thrombosis (91.3% of patients with TEVAR versus 19.4% of those who received medical treatment alone -P < 0.001 -). These results are in line with our suggestions confirming that optimal medical therapy is the treatment of choice of type B AAD with stable clinical conditions reserving a more aggressive management as TEVAR in patients with severe complications and to stabilize aortic wall so determining the false-lumen thrombosis.

We also analyzed the late mortality aorta-related with 3 late deaths in the OS group so demonstrating a clear and a better survival rate in the TEVAR group. Nevertheless we do not consider this result a statement especially evaluating the limits represented by the low number of patients enrolled.

There are still controversies regarding the optimal surgical strategy in patients with type B AAD. Since 1993 we perform in all descending aorta operations femoro-femoral bypass in normothermia to guarantee distal organ perfusion and placing the proximal aortic clamp between the left carotid artery and the left subclavian artery or just below the left subclavian artery. Shimokawa and colleagues [20] presented their results in the treatment of acute type B dissection with distal arch replacement and left heart bypass on mild hypothermia showing reduction in the incidence of postoperative fatal complications. The use of hypothermic circulatory arrest avoiding aortic clamping between left carotid artery and left subclavian artery improves surgical results but a longer circulatory arrest determines a poor outcome [6]. Lansman and coworkers [21] noticed that in their series of 34 patients undergoing surgery for type B AAD the use of hypothermic circulatory arrest was reserved in 16 cases with no operative mortality and low incidence of paraplegia. According to Shimokawa and colleagues [20] and Lai and coworkers [22] we firmly believe that proximal clamp can be safely placed in a preferred fashion in all cases when dissection does not involve the aortic arch. It is obvious that further investigations will confirm this statement.

Moreover, our results demonstrate high incidence of paraplegia/paraparesis in the OS group probably due to the variable "acute dissection" itself as reported by Panneton and Hollier [23] and Coselli and coworkers [24] who conclude that only acute dissection increases the risk of this neurologic complication and suggest critical intercostal artery reattachment and atriodistal bypass as safe procedures with predictable results.

Our data have suggested early encouraging results for TEVAR vs OS in patients with type B AAD; however the comparison at follow-up is not clearly defined with a cumulative survival at 1 (93% vs 86%), 3 (84% vs 71%) and 8 (69% vs 57%) years higher in terms of percentage in the endovascular group.

### Study limitations

There are some limitations of this study primarily related to its retrospective and observational characteristics including our evolving experience with TEVAR with limitations and benefits, and considering that we have used two different techniques during two different periods. In fact one of the indications to include patients in the TEVAR group was the presence of important comorbidities together with technical criteria already explained. Moreover we are conscious that the size of the cohort was small and not randomized, the two groups of patients are not entirely comparable and the time-related enrolment of patients was quite long. Further randomized, controlled studies are needed to address the best therapeutic strategy for complicated type B AAD and factors associated with optimal short- and long-term outcomes.

## Conclusions

In summary, this study shows that TEVAR may be considered as therapeutic option in these series of complicated patients with low early mortality and low incidence of postoperative complications. Moreover shorter postoperative length of stay may be considered no negligible feature in the TEVAR group compared to patients undergoing conventional surgery.

However, after all these reflections, the final act of this study was not to assert the superiority of endovascular procedure over conventional surgery nor to consider it as an alternative to surgical repair but to define further knowledge on the role of endovascular stent-graft repair in the treatment of all descending aorta diseases including type B AAD. We believe that our work contains meaningful information that can represent the basic rationale for future larger studies in larger populations.

## Competing interests

The authors declare that they have no competing interests.

## Authors' contributions

**PM **contributed to conception and design of the paper, analysis and interpretation of data, revision of the manuscript. **FO **and **SZ **carried out data. **AR **and **CI **participated in the revision of the manuscript critically for important intellectual content. All Authors read and approved the final manuscript.
